# Proteomics identification and characterization of MbovP730 as a potential DIVA antigen of *Mycoplasma bovis*

**DOI:** 10.18632/oncotarget.22265

**Published:** 2017-11-02

**Authors:** Farhan Anwar Khan, Gang Zhao, Yusi Guo, Muhammad Faisal, Jin Chao, Xi Chen, Chenfei He, Harish Menghwar, Rahim Dad, Muhammad Zubair, Changmin Hu, Yingyu Chen, Huanchun Chen, Zhang Rui, Aizhen Guo

**Affiliations:** ^1^ The State Key Laboratory of Agricultural Microbiology, Huazhong Agricultural University, Wuhan 430070, People’s Republic of China; ^2^ College of Veterinary Medicine, Huazhong Agricultural University, Wuhan 430070, People’s Republic of China; ^3^ Department of Animal Health, Faculty of Animal Husbandry and Veterinary Sciences, The University of Agriculture, Peshawar, Khyber Pakhtunkhwa 25120, Pakistan; ^4^ Key Laboratory of Development of Veterinary Diagnostic Products, Ministry of Agriculture, Wuhan 430070, People’s Republic of China; ^5^ International Joint Research and Training Centre for Veterinary Epidemiology, Hubei Province, Huazhong Agricultural University, Wuhan 430070, People’s Republic of China; ^6^ Key Laboratory of Agricultural Animal Genetics, Breeding and Reproduction, Education Ministry of China, Huazhong Agricultural University, Wuhan 430070, People’s Republic of China

**Keywords:** bovine, mycoplasma bovis, membrane proteins, immunoproteomics, qPCR

## Abstract

*Mycoplasma bovis* (*M. bovis*) is an important pathogen of cattle. An attenuated live vaccine has recently been developed by this laboratory. However, an effective assay for the differentiation of infected from vaccinated animals (DIVA) is still lacking. Therefore, a comparative immunoproteomics study of the membrane and membrane associated proteins (MAPs) of *M. bovis* HB0801 and its attenuated strain (*M. bovis*-150) was aimed to identify potential antigens for DIVA assay. Triton-X-114 fractionated liposoluble proteins of both the virulent and attenuated strains were separated with 2-DE and proteins reacting with sera against the virulent *M. bovis* strain were detected by MS. A total of 19 differently expressed proteins were identified by MS, among them twelve proteins were detected by MALDI-TOF MS and seven antigenic proteins were identified by short-gun LC-MS/MS. Furthermore, these findings were confirmed at mRNA level by qRT-PCR. The results demonstrated that a putative lipoprotein encoded by functionally unknown gene Mbov_0730 (MbovP730) is a sensitive and specific antigen for DIVA assay. MbovP730 is absent in *M. bovis*-150 confirmed with Western blot assay and also didn’t cross-react with other antisera against common pathogens including infectious bovine rhinotracheitis virus and bovine viral diarrhea virus by iELISA. Thereby rMbovP730-based iELISA was established. For clinical samples, this ELISA provided a sensitivity of 95.7% (95% CI: 90.4%, 98.2%) and specificity was 97.8% (95% CI: 88.4%, 99.6%). Antisera from vaccinated calves (*n =* 44) were found negative with rMbovP730 based iELISA, while positive with assays based on whole cell proteins of *M. bovis*-150 and *M. bovis* HB0801, respectively. In conclusion, this study identified the differential antigen MbovP730 between virulent and attenuated strains and established rMbovP730-based iELISA as a new DIVA method.

## INTRODUCTION

*Mycoplasma bovis* (*M. bovis*) belongs to *Mollicutes*, a class of simple self-replicating organism characterized by a small genome and absence of a cell-wall. It is a successful pathogen of cattle, characterized by multiple diseases such as pneumonia, mastitis, arthritis, keratoconjunctivitis, otitis, meningitis, orchitis, vesiculitis, infertility and abortion leading to huge economic losses to global cattle industry [1–4]. In Europe, it is responsible for about a quarter of all calf pneumonia with annual losses estimated at 140 million Euros [2]. The economic impact of *M. bovis* in the USA is similar to that of Europe due to mastitis and respiratory infections [2, 5]. In China, *M. bovis* was first identified in feedlot cattle with severe pneumonia by our laboratory in 2008 and was found causing more than 80% morbidity and on average 10%, sometime 60% mortality in diseased cattle population [6]. The exhaustive transportation of calves from northern to southern and central parts of the country is thought to be the main contributing reason of *M. bovis* related to disease epidemics in cattle [6].

*Mycoplasma bovis* pneumonia has quickly emerged as a serious threat to cattle industry in China, especially to beef industry, due to rapid expansion of the livestock industry, emerging resistance to formerly efficient antimicrobials [4], and a lack of protective vaccine, even though vaccine development is presently a focused research area [7]. More recently our laboratory has developed a live protective vaccine for the prevention of *M. bovis* related disorders in cattle by using an attenuated strain derived from wild type strain *M. bovis* HB0801 after 150 *in vitro* passages [5, 8, 9] namely *M. bovis*-150. However, research work on this *M. bovis* live vaccine is still limited, and an efficient serodiagnostic assay for the differentiation of infected from vaccinated animals is still needed. In this prospective, we recently established a *M. bovis* antibody avidity assay based on sodium thiocyanate (NaSCN) competitive indirect enzyme linked immunosorbent assay (iELISA) using whole cell proteins of *M. bovis* [9] for the differentiation of animals infected with *M. bovis* from vaccinated animals (DIVA). The sensitivity, specificity and simplicity remains to be improved.

Complete genome sequences of *M. bovis* strains [8, 10, 11] paved the way for the identification of specific proteins for differentiation between virulent and vaccine strains based on proteomic studies. However, it is usually reported that 2-DE has some limitation in separation of very hydrophobic and basic proteins, which are mostly abundant in the membrane of mycoplasma [1, 12, 13]. Indeed, membrane and membrane linked proteins are inadequately identified in 2-DE analysis of whole cell proteins of mycoplasma [14, 15]. However, Triton X-114 (TX-114) fractionation could improve the resolution of membrane associated proteins on 2-DE gel, since it was established to selectively improve the resolution of liposoluable proteome [16, 17, 18, 19, 20]. Triton X-114 fractionation followed by 2-DE is still the preferable approach for the characterization of the membrane and membrane associated proteins and for investigation of differential expression of membrane protein among bacterial strains either virulent or attenuated by proteomics [19, 20, 21, 22]. In addition, immunoproteomics by combining proteomics approaches like 2-DE and MALDI-TOF MS and immunobloting assay has been increasingly and successfully used to identify immunological antigens of several mycoplasma species [14, 15, 20, 23, 24].

Currently no comparative 2-DE immunoproteome map of the virulent *M. bovis* HB0801 and attenuated vaccine strain *M. bovis*-150 is available. Therefore, comprehensive exploratory studies on the membrane and membrane associated proteins (MAPs) of such infectious agent and its attenuated strains might offer new and attractive approach on its biology, and produce practical information for the improvement of control strategies, and diagnostics. It is well known that membrane and MAPs are playing an essential role in the colonization and survival of mycoplasmas within their hosts due to lack of a cell wall [13]. Besides other proteins, a large numbers of lipoproteins linked to the cytoplasmic membrane have been reported, majority of these lipoproteins are thought to be exposed to the cell surface. However, only a few lipoproteins have previously been determined the properties related to virulence or antigenicity [14, 25]. In addition, due to early exposure of lipoproteins to the host immunity, a very conserved, sensitive, and specific antigenic membrane or MAPs could be the best possible alternative for establishment of novel DIVA serodiagnostics.

This study was designed to detect antigenic membrane proteins that differentially expressed between the virulent *M. bovis* HB0801 and attenuated vaccine strain *M. bovis*-150 by immunoproteomics (2-DE, Western Blotting, MS). This study identified some new antigens and determined MbovP730 as a specific and sensitive DIVA antigen. An indirect ELISA (iELISA) based on recombinant MbovP730 (rMbovP730) was established and evaluated as a potential DIVA serological assay.

## RESULTS

### Immunoproteomics of the MAPs of virulent and attenuated strains

The differential PCR confirmed both the virulent HB0801 and attenuated *M. bovis*-150 strains to be correct by generating an amplicon of 238 bp, a unique band of specie specific *uvrC* gene for *M. bovis*-150 strain and additional band of 146 bp, unique to gene Mbov_0732 for the virulent HB0801 strain.

**Figure 1:**
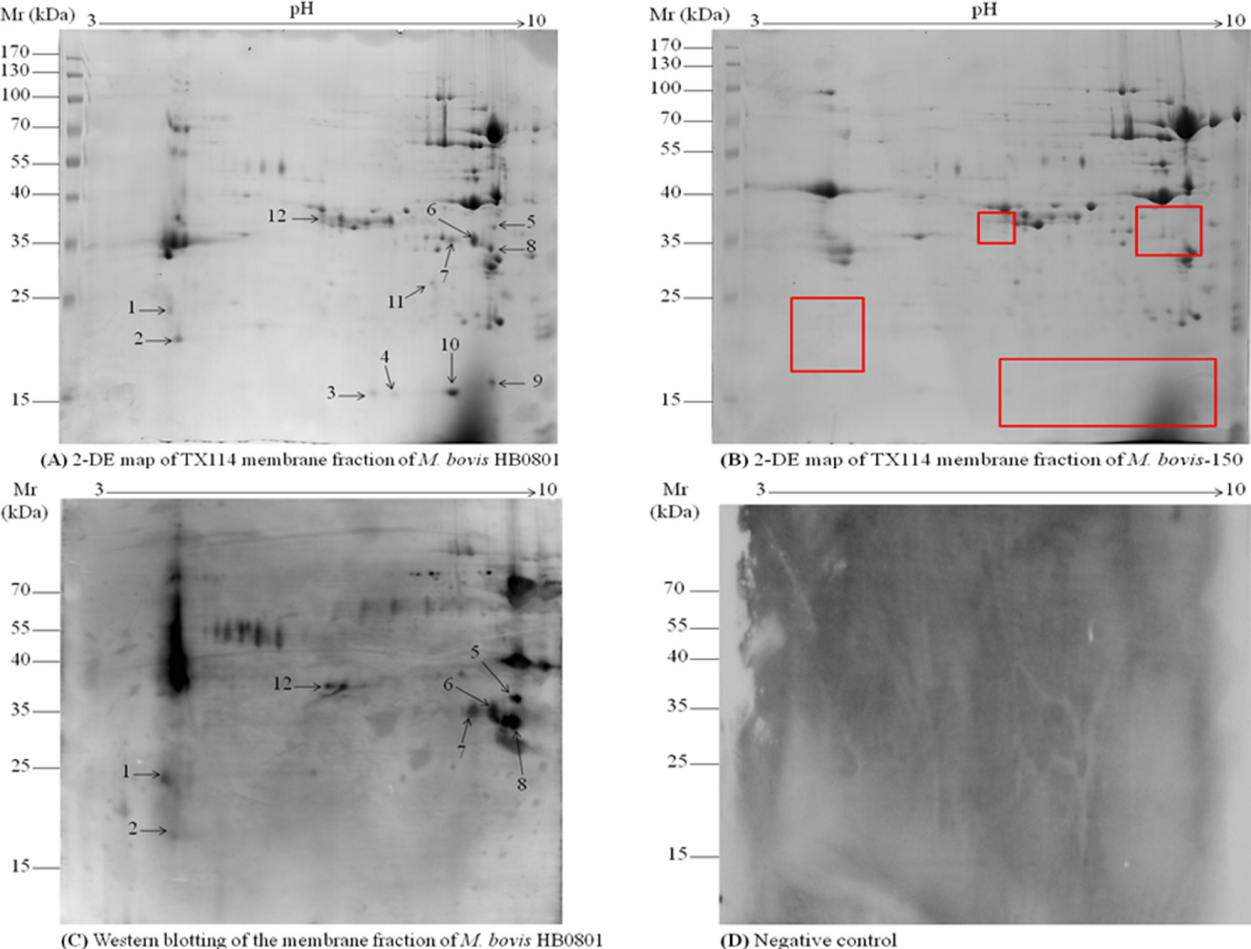
2D gel electrophoresis of TX-114 membrane fractions of *M. bovis* and immunoblotting assay TX-114 membrane fractions of *M. bovis* HB0801 wild type and its attenuated strain *M. bovis*-150 were extracted and subject to be 2D gel electrophoresis. Isoelectric points are indicated on top and molecular weight markers are on the left in kDa. (**A**) Analysis of HB0801 membrane proteins by 2-DE. (**B**) 2-DE analysis of *M. bovis*-150 membrane fraction. (**C**) Immunoblotting pattern of HB0801 membrane proteins by using antisera derived from calves experimentally infected with HB0801. (**D**) Negative control, no background signal was observed with sera from the infected animals at day 0 and sera from the uninfected animals in the experiment. The 12 spots identified down-regulated are indicated on the 2-DE gel of the HB0801 proteins. The red-boxes indicated the down-regulated protein spots position on 2-DE gel of *M. bovis*-150 proteins.

The MAPs extraction was carried out from the cultures under standardized conditions and the TX-114 membrane fractions of HB0801 and *M. bovis*-150 strains were separated on a wide-range 17 cm non-linear (pH 3–10 NL) IPG strips. The comparative analysis of the proteins spots on the 2-DE gel between MAPs of virulent and attenuated strains were performed. Among significantly down-regulated spots of *M. bovis*-150 MAPs, 12 were selected for further analysis (Figure 1A, 1B). In addition, three independent experiments show a high reproducibility.

Furthermore, TX-114 membrane fraction of *M. bovis* HB0801, separated by 2-DE and blotted onto PVDF membrane, was subjected to Western blot analysis. Proteins in only seven spots among the twelve differentially expressed spots (Figure 1C) were found reactive with pooled antisera from 20 experimentally infected calves that included spots # 1, 2, 5, 6, 7, 8, and 12 (Tables 1, 2) in the 2-DE map of *M. bovis* HB0801 membrane proteins (Figure 1C). As anticipated no background signals were observed with the negative sera collected from the animals at day 0 after infection and sera from the uninfected animals in the experiments (Figure 1D).

**Table 1:** Antigenic proteins identified by MALDI-ToF MS differentially expressed between virulent *M. bovis* HB0801 and vaccine strain *M. bovis*-150

Spot No.	Gene	Protein	NCBI ID	Mr (kDa)	pI	Protein Score C.I %	Peptide Count	PSORTb Localization	PSORTb Probability	COG^**^
6	Mbov_0070	Putative transmembrane protein	gi|392051070|	29.19	8.33	76.091	8	Cytoplasmic Membrane	10.00	-
8	Mbov_0130	Putative transmembrane protein	gi|392051130|	26.3	9	100	9	Unknown	-	S
5	Mbov_0306	Phosphonate ABC transporter substrate-binding protein	gi|392051299|	51.5	9.04	99.997	10	Cytoplasmic Membrane	9.97	P
12	Mbov_0312	Alcohol Dehydrogenase	gi|392051305|	37.5	6.51	100	5	Cytoplasmic	9.97	E, R
8	Mbov_0512	Hypothetical protein	gi|392051491|	36.4	8.93	100	10	Cytoplasmic	8.96	-
6-7	Mbov_0593	Cobalt/nickel ABC transporter permease	gi|392051572|	35.4	8.73	100	11	Cytoplasmic Membrane	8.78	P
5	Mbov_0628	50S ribosomal protein	gi|392051604|	35.3	10.24	99.993	6	Cytoplasmic	9.26	J
8	Mbov_0693	Putative transmembrane protein	gi|392051667|	302.4	8.41	76.635	40	Extracellular	8.91	-
8	Mbov_0702	Transcriptional accessory protein	gi|392051676|	81.4	8.56	74.381	18	Cytoplasmic	8.91	K
6-7-8	Mbov_0796	Variable surface lipoprotein (Vsp)	gi|392051769|	31.5	9.01	100	10	Unknown	-	-
5-8	Mbov_0798	Variable surface lipoprotein L (Vsp L)	gi|392051771|	30.4	9.04	100	10	Unknown	-	-
7	Mbov_0845	FtsH cell division protease	gi|392051814|	75.2	9	68.482	16	Cytoplasmic Membrane	10.00	O

^**^ O, Posttranslational modification protein; J, translation, ribosomal structure and biogenesis; P, Inorganic ion transport and metabolism; K, Transcription; E, amino acids transport and metabolism; R, General function prediction only; S, Glycerophospholipid metabolism.

**Table 2:** Antigenic proteins identified by LC-MS/MS differentially expressed between virulent *M. bovis* HB0801 and vaccine strain *M. bovis*-150

Spot No.	Gene	Protein	NCBI ID	Mr (kDa)	pI	Peptide Count	PSORTb Localization	PSORTb Probability	COG^**^
1	Mbov_0305	Putative trans-membrane protein	gi|392051298|	68.71	4.88	3	Cytoplasmic	8.96	-
1	Mbov_0495	Mg2+ Transport protein	gi|392051476|	54.13	4.74	2	Cytoplasmic Membrane	9.80	P
1	Mbov_0730	Putative Lipoprotein	gi|392051003|	33.5	9.17	2	Unknown	-	-
1	Mbov_0732	Putative Lipoprotein	gi|392051706|	38.03	5.56	3	Unknown	-	-
1–2	Mbov_0797	Variable Surface Protein-K (*VSP*-K)	gi|392051770|	34.60	5.02	2	Unknown	-	-
1	Mbov_0838	Putative Lipoprotein	gi|392051808|	50.29	6.94	1	Periplasmic	9.83	-
1	Mbov_0856	Putative Lipoprotein	gb|AFN86082.1|	6045.14	9.9	1	Unknown	-	-

^**^ P, Inorganic ion transport and metabolism.

### Identification of potential DIVA diagnostic antigens by MAILDI-ToF MS

On the basis of immunoproteomics analysis, prominent antigenic protein spots were extracted from the 2-DE gels and prepared for mass spectrometry (MS) analysis. MALDI-TOF MS analysis revealed the presence of proteins in five protein spots (Spots # 5, 6, 7, 8, and 12) (Figure 1A) corresponding to a total of 12 proteins. Nine proteins were recognized in one spot, while three proteins including variable surface protein-L (*VSP*-L) and cobalt/nickel ABC transporter permease were characterized in two spots, and a member of *VSP* family encoded by CDS Mbov_0796 was identified in three spots suggesting post-translational modifications, such as chemical modification and proteolytic cleavage, of these proteins (Table 1). The identity percentage, isoelectric point, molecular mass, identification score, and protein score of these proteins were presented in Table 1.

Among these 12 antigenic proteins, four proteins were predicted as cytoplasmic with PSORTb analytical tool, five were predicted MAPs, while the remaining three proteins were unknown to PSORTb probability (Table 1). According to the clusters of orthologous groups (COG) of functional classification system, seven of the twelve proteins were assigned to a broad range of categories. The phosphonate ABC transporter substrate-binding protein, and cobalt/nickel ABC transporter permease protein were found to be involved in inorganic ion transport and metabolism (P); 50S ribosomal protein in translation, ribosomal structure and biogenesis (J); ftsH cell division protease and posttranslational modification of protein (O); putative transmembrane protein and conserved hypothetical protein in glycerophospholipid metabolism (S);transcriptional accessory protein in transcription (K); and ADH in amino acid transport and metabolism (E); while seven others are hypothetical proteins with unknown functions (Table 1).

### Identification of antigenic proteins by LC-MS/MS analysis

A combination of 2-DE and LC-MS/MS proteomic analysis was further employed to identify proteins in the two antigenic protein spots left unidentified by MALDI-TOF MS. Remarkably, the analysis recognized seven additional antigenic proteins (Table 2) in the two protein spots (Spots # 1 and 2) down-regulated on the 2-DE map of *M. bovis*-150 proteins (Figure 1A, 1B).

Among these seven proteins, mostly functionally unknown, a putative trans-membrane protein (encoded by CDS Mbov_0305) was predicted as cytoplasmic with PSORTb analytical tool, two proteins were predicted MAPs including Mg^2+^ transport protein, and putative lipoprotein (encoded by CDS Mbov_0838), while four proteins were unknown to PSORTb probability (Table 2). The COG analysis characterized the Mg^2+^ transport protein involved in the inorganic ion transport and metabolism (P). Importantly, two proteins including putative lipoproteins encoded by CDS Mbov_0730 and 0732, found deleted in the genome of the attenuated vaccine strain *M. bovis*-150 in our laboratory [26] by whole genome sequence analysis, were also identified antigenic (Spot # 1) in the *M. bovis*-HB0801 (Figure 1A, 1B, 1C).

### Validation of antigen expression difference between virulent and attenuated *M. bovis* strains

**Figure 2:**
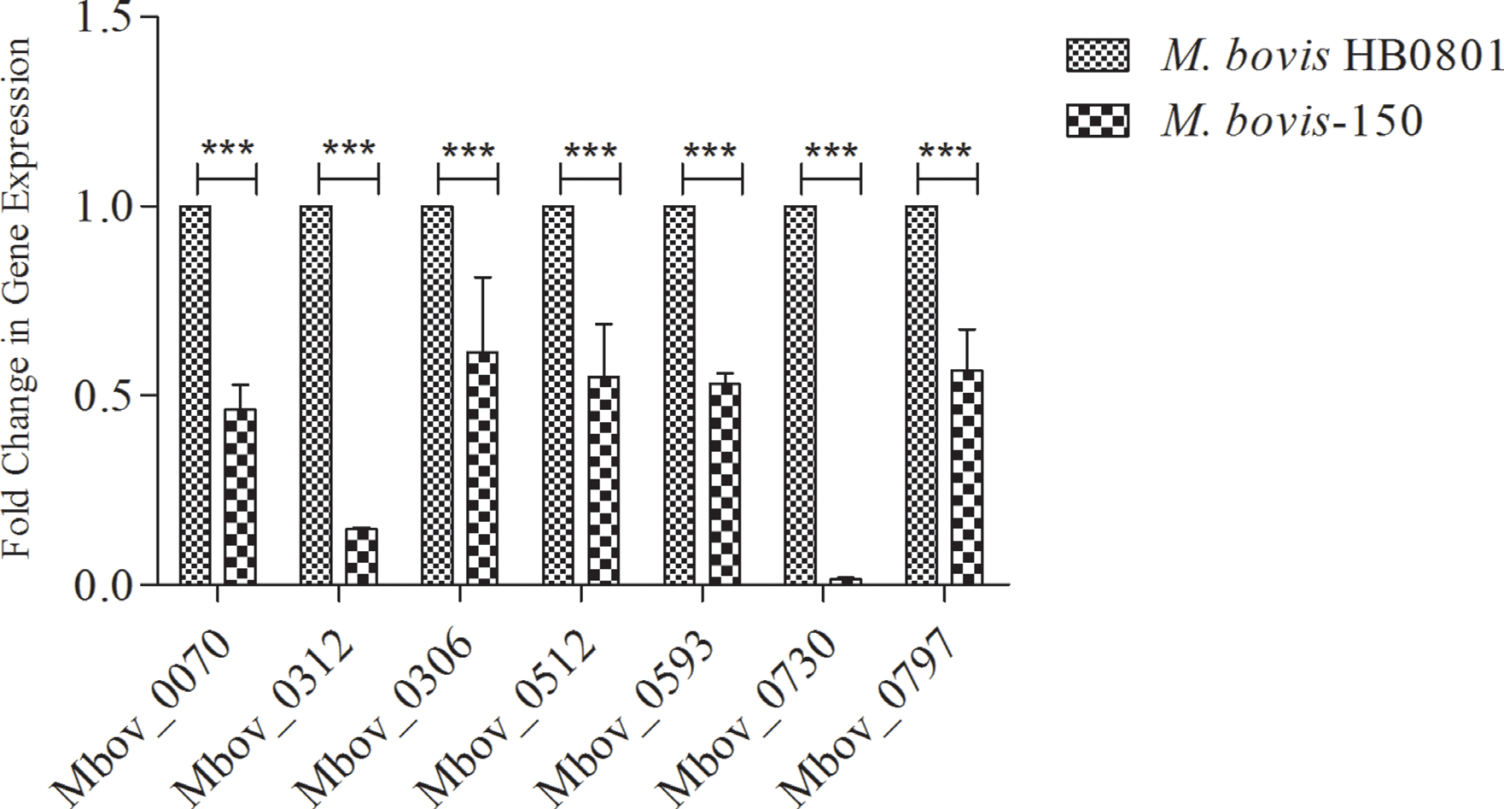
Verification of down-regulated proteins in the attenuated strain *M. bovis*-150 RT-qPCR analysis revealed down-regulation of several genes (Mbov_0070, 0312, 0306, 0512, 0593, 0730, 0797) during attenuation of *M. bovis* HB0801. Data are presented as mean ± SD. Representative image was selected from three independent experiments performed in triplicate. β-actin was used as the internal control.

Seven proteins were predicted to have a large number of T- and B-cell epitopes (Tables 1, 2) (Figure 2). For the confirmation of immunoproteomics findings, qRT-PCR was performed. The result demonstrated that the gene expression levels of those seven antigenic proteins showed the same tendency of changes as the quantitative 2-DE-MS results (Figure 2). These seven antigenic proteins (putative transmembrane protein encoded by CDS Mbov_0070 (Spot # 6), phosphonate ABC transporter substrate-binding protein (Spot # 5), ADH (Spot # 12), hypothetical protein encoded by CDS Mbov_0512 (Spot # 8), cobalt/nickel ABC transporter permease protein (Spots # 6,7), putative Lipoprotein encoded by CDS Mbov_0730 and *VSP-*K encoded by CDS Mbov_0797 (Spots # 1,2) were significantly expressed only in the virulent and down-regulated (*p* < 0.01) in the attenuated strain of *M. bovis* at mRNA level.

### Cloning, and expression of MbovP730

**Figure 3:**
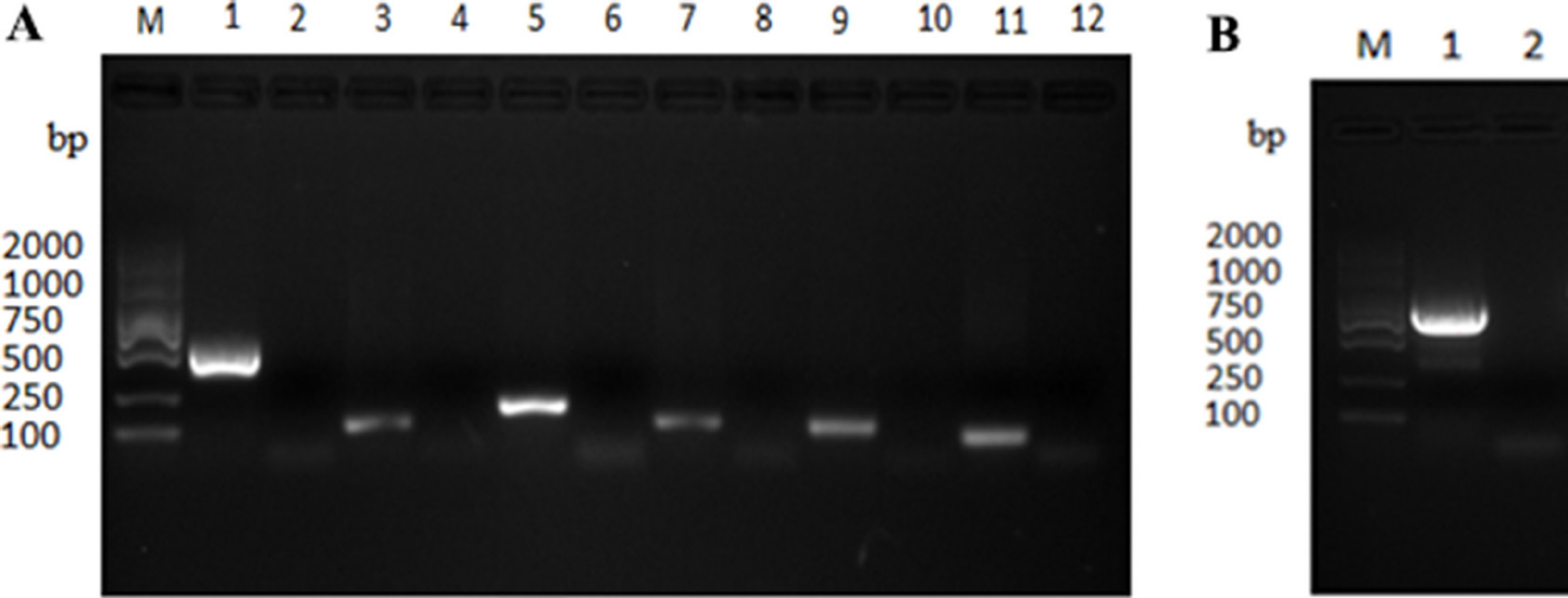
Cloning of *M. bovis* Mbov_0730 and site directed mutagenesis with overlap extension PCR (**A**), Six fragments of Mbov_0730 was cloned by PCR. (**B**) the whole gene was cloned using six fragments of Mbov_0730 as a template. “M” indicated gene marker (2000 bp), whereas lanes; 1,3,5,7,9,11 in Figure 5A indicated six fragments (509 bp, 96 bp, 169 bp, 107 bp, 105 bp, 73 bp respectively) of Mbov_0730, and lanes; 2,4,6,8,10,12 indicated negative control. Lane “1” in Figure 5B indicated amplification of complete gene Mbov_0730 (867 bp).

**Figure 4:**
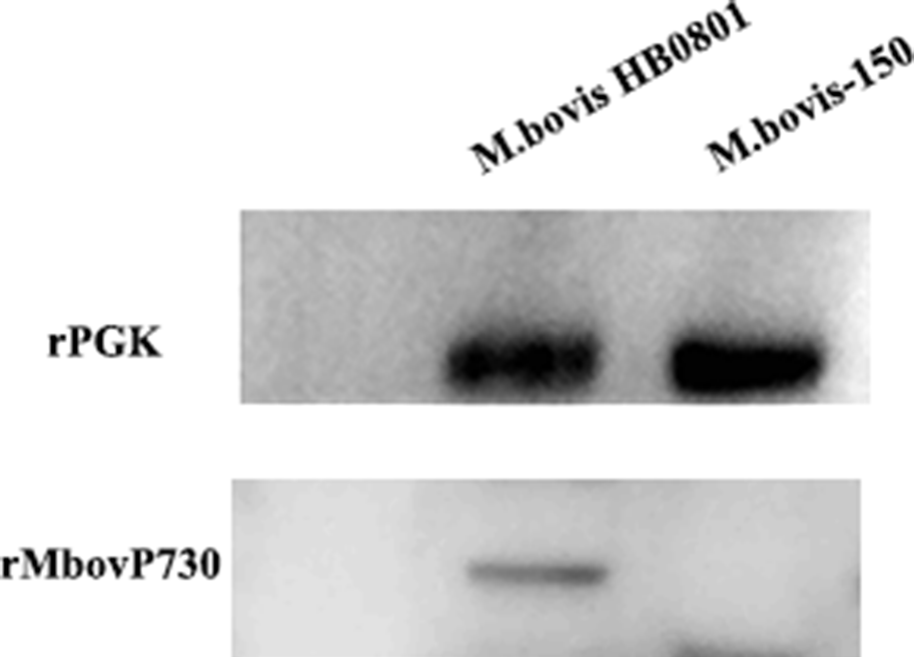
Expression of the MbovP730 in *M. bovis* strains The polyclonal antibodies were used to verify the existence of Mbov_0730 in the HB0801 and *M. bovis*-150. PGK was used as a positive control.

To circumvent the specific translational barrier of the UGA codon between mycoplasmas and *E. coli* for expression of Mbov_0730 gene, the mutagenesis of *M. bovis* UGA to UGG was performed by overlapped extension PCR to ensure encoding of tryptophan for Mbov_0730 in *E. coli*. A total of eight sites were mutated, using primers listed in Table 3, in a total of six overlapped fragments of gene Mbov_0730 (Figure 3A). The mutated segments were ligated to a complete gene, and the full gene was subsequently cloned into pET-30a (+). The mutagenesis and cloning was confirmed to be correct by sequencing and the alignment assay (Figure 3B). As a result, the cloned gene had a 100% of similarity with the published sequence of HB0801 at nucleotide level except above modifications, and was inserted into the vector correctly. Then the expression of recombinant proteins induced with isopropyl-b-D-1-thiogalactopyranoside (IPTG) was confirmed with SDS-PAGE (Figure 4).

**Table 3:** Homologues of MbovP730 of *M. bovis* HB0801 in sequenced *M. bovis* strains (NCBI data base)

Pathogens	Mnemonics	Predicted gene function	Identity %
*M. bovis* PG45	MbovPG45_0367	lipoprotein	90
*M. bovis* Hubei-1	MMB_0691	lipoprotein	99
*M. bovis* CQ-W70	K668_03450	hypothetical protein	100
*M. bovis* NM 2012	AAV31_03620	hypothetical protein	100
*M. bovis* 08M	B0W43_03720	hypothetical protein	100

### Specificity of the recombinant MbovP730

The monospecific antiserum against the recombinant MbovP730 (rMbovP730) were raised in Balb/c mice to determine its specificity in *M. bovis* strains. The titer of mouse monospecific antiserum against rMbovP730, which was determined with an iELISA, was 10^6^. Western blot analysis with this mouse antiserum against rMbovP730 revealed specific expression of the respective protein in *M. bovis* HB0801 (Figure 4), whereas lack of immunoblot signal in *M. bovis*150 further confirmed the deletion of MbovP730 gene (Figure 4). Furthermore, MbovP730 was found conserved in all sequenced strains of *M. bovis* (Table 3) by using NCBI GenBank database. Accordingly, homologues of MbovP730 of *M. bovis* HB0801 were also found in all sequenced Chinese strains of *M. bovis* (Table 4).

**Table 4:** Conservation of the MbovP730 in the sequenced Chinese strains of *M. bovis*

Country	Provinces	Cities/Villages	Isolate name	Year of isolation	Specimen	Identity %
China (*n* = 29)	Hubei (*n =* 14)	Yingcheng	HB0801	2008	Lung	100
		Suizhou	Hubei-1	2008	Lung	99
		Jingshan	JS1075-NHD0955	14/05/2008	Lung	100
		Suizhou	SZ-NHD0960	07/06/2008	Lung	100
		Ezhou	1834-NHD0953	10/06/2008	Lung	100
		Ezhou	EZ-3-NHD0947	10/07/2008	Lung	100
		Ezhou	EZ-8-NHD0962	10/07/2008	Lung	100
		Ezhou	EZ-2-NHD0986	10/07/2008	Lung	100
		Xinzhou	XZ-1-NHD0981	11/07/2008	Lung	100
		Xinzhou	XZ-2-NHD0946	11/07/2008	Lung	100
		Hongan	NNH-NHD0956	01/05/2010	Throat	100
		Daye	DY-NHD0963	23/07/2010	Lung	100
		Tongshan	TY-120615-NHD0952	15/06/2012	Lung	100
		Jiangxia	JX-NHD0966	15/07/2012	Lung	100
	Anhui (*n* = 1)	Bozhou	BZ-NHD0982	10/06/2008	Lung	100
	Fujian (*n* = 2)	Xianmen	XM- NHD0959	17/10/2009	Lung	100
		Xinanmen	XMrengong -NHD0985	11/10/2012	Lung	100
	Hunan (*n* = 1)	Lianjiang	LJ1225-NHD0945	22/12/2009	Lung	100
	Henan (*n* = 5)	Yanling	YL-NHD0941	25/02/2009	Lung	100
		Kaifeng	KF- NHD0944	10/10/2009	Lung	100
		Yanling	YL0724-NHD0957	12/11/2009	Lung	100
		Yanjing	YJ0719-NHD0958	03/02/2012	Lung	100
		Yanling	YLrengong -NHD0968	11/10/2012	Lung	100
	Inner Mangolia (*n* = 1)	Neimeng Yuliang	YL2086	19/07/2012	Lung	100
	Jiangxi (*n* = 1)	Xinyu	JXXY-NHD0943	06/10/2012	Lung	100
	Guangzhou (*n* = 2)	Shenzhen	SZ- 0527- NHD0948	27/05/2012	Lung	100
		Shenzhen	SG-NHD0983	01/04/2013	Lung	100
	Shandong (*n* = 1)	Shandong	SD-130626-NHD0969	24/06/2013	Lung	100

### Performance of rMbovP730-based iELISA

**Figure 5:**
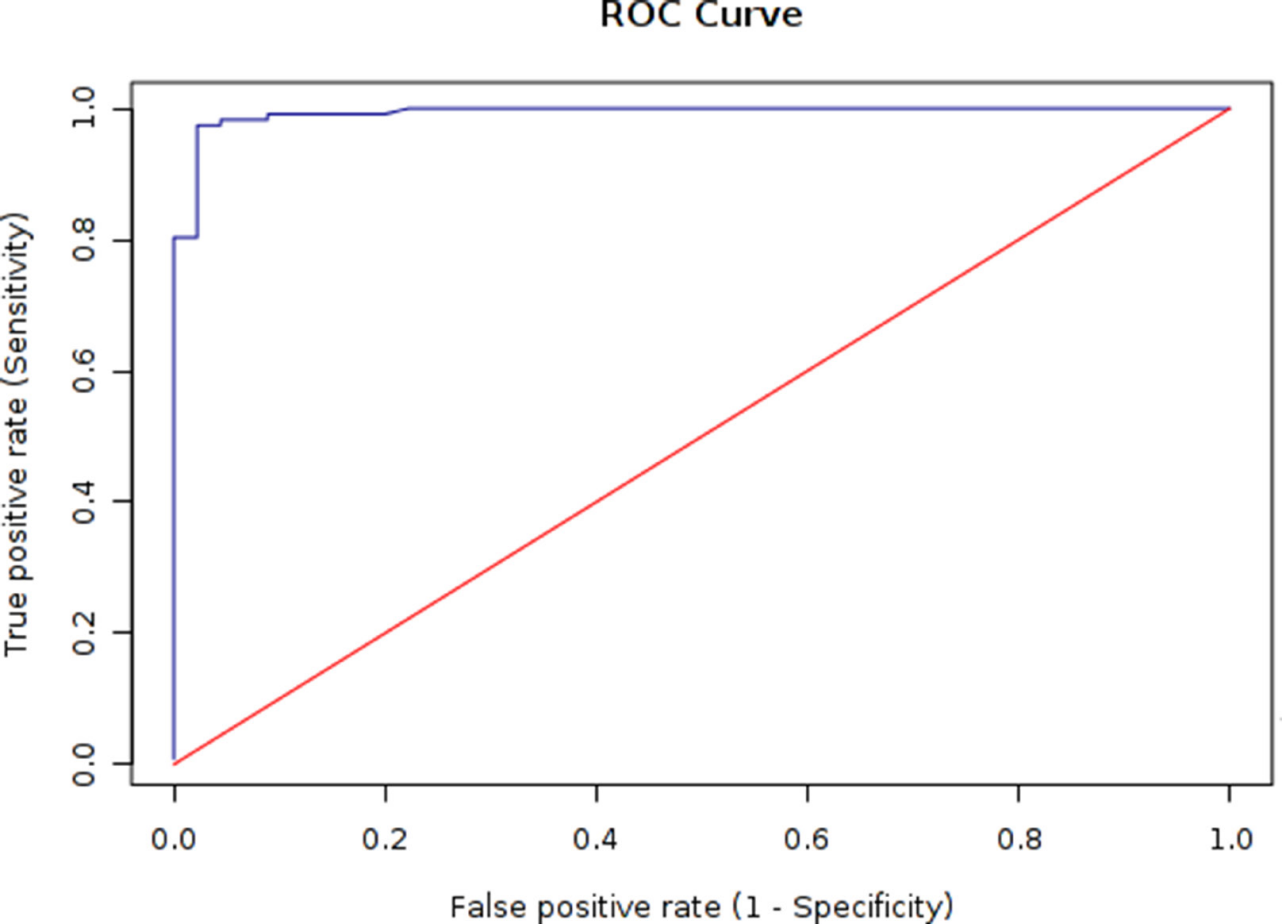
ROC analysis of the rMbovP730-based iELISA According to the ROC curve, cut-off value of an iELISA was determined to be 0.497 with a sensitivity of 95.7% and specificity of 97.8%.

Based on the presence of MbovP730 in *M. bovis* HB0801 and its deletion in *M. bovis*-150 and its antigenicity, an indirect ELISA based on rMbovP730 was developed to check the probability of rMbovP730 as a specific and sensitive antigen for DIVA serological assay. The optimal concentration of the coating antigen (rMbovP730) and optimal dilution of sera were determined separately for the iELISA by using the highest OD_630_ ratios (P/N) of positive (P) to negative sera (N). When the P/N ratio was set to be 4.02, the optimal antigen (rMbovP730) and serum dilutions were determined to be, respectively, 50 ng/well and 1:200, and the secondary antibody dilution was optimized to be 1:20000 (v/v). The cut-off value (OD_630_) was calculated to be 0.497 by ROC analysis. The natural infection and un-infection cases was determined by the *M. bovis* isolation and characterization which was used as the gold standard test, the diagnostic sensitivity of this rMbovP730-based iELISA was 95.7% (95% CI: 90.4%, 98.2%) and the specificity was 97.8% (95% CI: 88.4%, 99.6%) respectively for test of the sera from the naturally infected (positive) and uninfected (negative) cattle, (Tables 5, 6, and Figure 5). For the sera from experimentally infected and negative control calves, diagnostic sensitivity of rMbovP730 based iELISA was 100% (20/20) and specificity was 100% (8/8). The estimated Kappa (k) value between the gold standard and rMbovP730 based iELISA was 0.897 showing a high agreement between them. In addition, rMbovP730 based iELISA detected antisera from vaccinated calves (*n* = 44) to be negative, while assays based on whole cell proteins of M. bovis-150 and M. bovis HB0801 detected the same antisera to be positive, demonstrating the potential of this in-house assay as DIVA. The reference antisera against IBRV and BVDV were found non-reactive with iELISA based on rMbovP730.

**Table 5:** Cut-point results for target sensitivity of rMbovP730-based iELISA

Target Se	Cut-point	Sensitivity	Se Lower 95% CL	Se Upper 95% CL	Specificity	Sp Lower 95% CL	Sp Upper 95% CL
0.999	0.347	1	0.968	1	0.778	0.637	0.875
0.995	0.347	1	0.968	1	0.778	0.637	0.875
0.99	0.385	0.991	0.953	0.998	0.911	0.793	0.965
0.98	0.395	0.983	0.94	0.995	0.956	0.852	0.988
0.95	0.497	0.957	0.904	0.982	0.978	0.884	0.996
0.9	0.594	0.906	0.839	0.947	0.978	0.884	0.996
0.8	0.717	0.803	0.722	0.865	1	0.921	1

**Table 6:** Performance of rMbovP730-based iELISA in the detection of *M. bovis* natural infection

		Diagnosed by *M. bovis* detection (Gold standard test)		
		Positive	Negative	Total
rMbovP730-based iELISA	Positive	111	1	112
	Negative	06	45	51
	Total	117	46	163

Furthermore, rMbovP730-based iELISA and an available commercial kit (BioX, Belgium) were employed to test in parallel serum samples from 20 experimentally infected calves and 117 naturally infected cattle. As a result all the infected animals (20/20) in experiments were determined to be positive with rMbovP730-based iELISA, whereas with the commercial kit, only 65% (13/20) infected calves in experiment were positive. In addition, when both methods tested the clinical serum samples, the sensitivity of iELISA was determined to be 95.7% (95% CI: 90.4%, 98.2%), whereas that of the commercial kit was only 33.3%. The serum samples verified negative for *M. bovis*-specific IgG by this iELISA were also found negative with the commercial kit (Table 7). The results recommended that the sensitivity of rMbovP730-based iELISA was significantly higher than the current commercial kit (*p* < 0.01).

**Table 7:** Comparison of rMbovP730-based iELISA and the commercial kit to detect *M. bovis* natural infection

		Commercial iELISA kit		
		Positive	Negative	Total
rMbovP730-based iELISA	Positive	39	72	111
	Negative	0	06	06
	Total	39	78	117

Furthermore, to determine the universal application value of the MbovP730 as DIVA antigen, we analyzed the conservation of MbovP730 in all sequenced strains of *M. bovis* (Table 3) and in Chinese strains of *M. bovis* (Table 4) and showed that MbovP730 is very conservative. This evidence, together with the above high sensitivity to the clinical samples supports that rMbovP730 based iELISA could detect most of prevalent strains of *M. bovis*.

## DISCUSSION

More recently our laboratory has developed a live attenuated vaccine using the *M. bovis*-150 strain and determined that it elicits protective immunity against challenge with virulent strain *M. bovis* HB0801 in experimental calves after a single intranasal administration [5]. That study demonstrated that primary immunisation provoked an induction of a low avidity IgG response, while natural infection stimulates a high avidity IgG response. Therefore, our laboratory established a novel IgG avidity test based on whole cell proteins, subsequent to the vaccine development, as a DIVA serological tool for *M. bovis* [9]. However, it would be possible that secondary vaccination might increase the IgG avidity and make this DIVA dependent on IgG avidity less sensitive. Therefore, in this study we used a combination of genomics and immunoproteomics to identify a more specific and sensitive biomarker of *M. bovis* for improvement of DIVA assay.

The *Mycoplasma bovis* membrane protein s were extensively separated with 2-DE, identified with immunoblotting, MALDI-TOF MS, LC-MS/MS, and confirmed with qRT-PCR the differentially expressed antigenic proteins. The MAPs, especially lipoproteins, are thought to be highly antigenic due to their presence on the surface and amino terminal lipoylated structure [26], but they usually do not contain membrane anchors, are associated with the membrane via their lipid tails and, consequently, may be partly lost during the extraction [15]. Therefore, we used TX-114 fractionation for the enrichment of lipoproteins as described previously [21, 22, 23, 24]. Our subsequent immunoproteome study detected a total of 19 antigenic protein using both MALDI-ToF and LC-MS/MS technologies. Coincidently, five antigens among our protein list, the *VSP* encoded by CDS Mbov_0796, *VSP-*K (encoded by CDS Mbov_0797), *VSP*-L (encoded by CDS Mbov_0798), mycoplasma immunogenic lipase A (*MilA*) (encoded by CDS Mbov_0693), and ADH have previously been identified in other *M. bovis* strains thereby lending support to the reliability of our findings. However none of them has been used as a DIVA diagnostic antigen. Remarkably, fourteen proteins were identified as new antigens and the majority of antigenic proteins were predicted to be MAPs.

Although highly antigenic, proteins of *VSP* family have virulence related properties but their hypervariablility in expression [7, 27, 28, 29, 30] makes the members of this family unsuitable for serodiagnostic assays. We identified antigenicity of alcohol dehydrogenase (ADH) (encoded by Mbov_0312) in *M. bovis* as reported previously [16], with the addition of its highly significant (*p* < 0.01) down-regulation in *M. bovis*-150 both at protein and mRNA levels. Further *in silico* analysis revealed the conservation of ADH in *M. bovis* with no homologue in other mycoplasmas. Although, ADH was found as potential candidate for DIVA assay, the specificity of ADH might be low because *M. bovis* has three isoenzymes (encoded by Mbov_0312, 0338, 0335), identified by genome sequence analysis. Although the surface localization and antigenic properties of cytoplasmic proteins like pyruvate dehydrogenase, elongation factor-thermo unstable (Ef-Tu), glyceraldehyde-3-phosphate dehydrogenase (GAPDH) have been demonstrated previously [16, 17, 18, 19], the identification of other cytoplasmic proteins, such as 50S ribosomal proteins, among the antigenic proteins of HB0801 strain after reacting with the sera of experimentally infected calves were never reported as antigenic in *M. bovis*. Immune-reaction of 50S ribosomal protein L4 might be due to its epitopes localized at the back of the *50s* subunit, as described in *E. coli* by immuno-electron microscopy elsewhere [31].

In our laboratory comparative whole genome sequence analyses of virulent strain *M. bovis* HB0801 [8] and attenuated vaccine strain *M. bovis*- 150 (NCBI Reference Sequence: NZ_CP007590.1) determined deletion of a large DNA fragment (14 kb) in the attenuated strain [32]. In this study we found the antigenicity of two lipoproteins encoded by Mbov_0730, and 0732. These genes belong to the deleted fragment in the attenuated strain. According to the published data [8], Mbov_0730 was found as a pseudogene of lipoprotein which contains frame shift mutation. But in this study we found its expression in HB0801 confirmed by LC-MS/MS. Currently there is no explicit understanding of their role in the virulence of *M. bovis*, however the differential expression and antigenicity of these two lipoproteins in this study open the way for the establishment of DIVA serological tool.

Among the 19 antigenic proteins discovered in this study, MbovP730 was determined as a most sensitive, specific and conserved antigen for DIVA assay. Thereby rMbovP730-based iELISA was established. The method could detect seroconversion at 14 days post experimental infection. In addition, the sensitivity of this iELISA was far higher than the commercial kit. To our best knowledge, it might be currently the most sensitive and specific method. Generally speaking, a bottleneck of the previous assays [9] was low specificity because of using *M. bovis* WCPs. The utmost advantage of this DIVA assay over the previous assay established by our laboratory [9], is the use of specific and sensitive recombinant antigen expressed differentially between virulent and attenuated strains of *M. bovis*. In addition, the universal application of this diagnostic method was supported by more recent work in our lab that Chinese isolates showed a high genetic homogeneity and determined that 97.7% of the isolates belong to the same genotype with the multilocus sequence typing (MLST) and pulsed field gel electrophoresis (PFGE) methods [33].

In conclusion, this study identified and characterized MbovP730 as a promising novel DIVA diagnostic antigen of *M. bovis* with comparative proteomics and immunological approaches. The iELISA based on rMbovP730 would be a very sensitive and specific DIVA serological assay and could be helpful in the elucidation of the magnitude of infection and the efficiency of vaccination in animal populations.

## MATERIALS AND METHODS

### Ethical statement about animal experiments

The protocols of the animal experiments involved artificial infection of *M. bovis* and antiserum production against the recombinant protein were approved by the Hubei Province Science and Technology Department, which is responsible for the ethics in animal experiments in Hubei, China (permit no. SYXK(ER) 2015-0084), in accordance to the approval of China Regulations for the Administration Affairs Concerning Experimental Animals (1988) and the Hubei Regulations for the Administration of Affairs Concerning Experimental Animals (2005). In addition the experiments on animals were performed under the strict supervision of Ethical Committee for Experimental Animals of Huazhong Agricultural University, Wuhan, China.

### Strains and culture conditions

*Mycoplasma bovis* HB0801 virulent strain, stored at the China Center for Type Culture Collection (CCTCC) (#M2010040), was isolated from lung lesions of a calf with pneumonia and identified by this laboratory [8]. An attenuated vaccine strain *M. bovis*-150 (CCTCC #M2011102) from HB0801 was generated, maintained [5] and sequenced by our laboratory. Strains were grown as described previously [9]. The primer sets used for the confirmation of *M. bovis* HB0801 and *M. bovis*-150 by PCR are listed in Table 8.

**Table 8:** List of primers used for the confirmation of *M. bovis* HB0801, and *M. bovis*-150 strain and in overlap extension PCR for site-directed mutagenesis

Primers	Sequence (5′→3′)	Remarks
*uvr*C-F	TAATTTAGAAGCTTTAAATGAGCGC	Confirmation of *M. bovis* specie
*uvr*C-R	CATATCTAGGTCAATTAAGGCTTTG	
*Mbov_0732-*F	AGCGACCAAAATACTAGAC	Confirmation of vaccine strain i.e*. M. bovis-*150
*Mbov_0732-*R	TCGTTGCCACTGTATTCA	
P730 1	ACGGAATTCATGCCTAATGATGGTTCA	*Eco*R I site
P730 2	ACAATTTTTTTGCCATTTTTTATACTGATCCAGTTTTTA	UGA to UGG
P730 3	ATAAAAACTGGATCAGTATAAAAAATGGCAAAAAAATTGT	
P730 4	GAAGAACAGTGAGCTTAATTCCTGTCCAATC	
P730 5	CACAAAGATTGGACAGGAATTAAGCTCACT	
P730 6	CAGCATACTTAATATTTGATGTATCCCATTCATTAAGGTT	
P730 7	AACCTTAATGAATGGGATACATCAAATATTAAGTATGCTG	
P730 8	ACGCTACCTCTAATTTTCCAATTTTTTAAAGACTGATCTA	
P730 9	TTTAAAAAATTGGAAAATTAGAGGTAGCGTTAATACCAAG	
P730 10	GTTTTCCAAGCGGTAGCCATATCCTT	
P730 11	AAAGGATATGGCTACCGCTTGGAAAAC	
P730 12	GCCAAGCTTTTATGCTTTTTTATAGTTATATAGCATATTT	*Hin*d III site

### Extraction of membrane associated proteins (MAPs)

The membrane associated proteins (MAP) of *M. bovis* were fractionated using Triton X-114 (TX-114) as demonstrated elsewhere [24]. In brief, after incubation for 36 h the culture of *M. bovis* HB0801 was washed with PBS. Bacterial pellet was resuspended in PBS containing 4% TX-114 (Sigma, USA), 1mM PMSF (Sigma, USA) and kept for 3–5 h at 4°C after lysis. The sample was then incubated 20 min at 37°C and then centrifuged 5 min at 7500 g to separate the two phases. The detergent phase was reconstituted with 1 mM PMSF in PBS and passed through a series of washing steps. The proteins were re-suspended in a modified lysis solution (8 M urea, 2 M thiourea, 4% CHAPS, 2% ASB-14, 60 mM DTT, 40 mM Tris- HCl pH 8.8). Protein concentration was measured with 2D quant kit (GE Healthcare, Sweden).

### Preparation of cattle antisera

Seventy-two clinically healthy local breed calves, 5–6 months of age, were first confirmed to be *M. bovis* free as described previously [9, 24]. These animals were randomly allocated to I of III groups. The 20 animals in Group I were inoculated with *M. bovis* HB0801 for three consecutive days, at a dose of 10^9^ cfu/calf, by intratracheal route. Forty four animals in group II were vaccinated with attenuated strain *M. bovis*-150 by intranasal route at a dose of 10^8^ cfu/calf only once. Group III included 8 calves that were exposed to sterile PPLO media as negative control group. Serum samples were collected from all the animals on day 0, 7, 14, 21, 28, 35 after infection/immunization. The animals were euthanized and necropsied for gross lesion evaluation at day 40 post infection/vaccination by using scoring system described previously [5]. Antibody titer of serum samples was measured by iELISA [9, 24]. Serum samples collected at day 0 before infection or immunization were used as negative control.

### Serum samples from the naturally infected and uninfected animals

The serum samples were collected from the 46 uninfected calves and 117 infected calves, determined by *M. bovis* isolation in this laboratory to be naturally infected with *M. bovis* at various feedlots of cattle in China. The disease was diagnosed as described previously [5, 9, 24]. All animal’s serum samples were stored at −80°C in our laboratory for further analysis.

### Two dimensional gel electrophoresis (2-DE)

For 2-DE, 380 μg and 750 μg of proteins were loaded onto analytical and preparative gels, respectively [20, 24]. The IPG strips were rehydrated for 16 h in 400 μl of rehydration buffer containing the protein samples. IEF was performed in five steps: 150V for 3 h, 300 V for 3 h, 1000V (gradient) for 6 h, 10000V (gradient) for 3 h, and 10000V for 60 kVh. The gel strips were equilibrated with 1% DTT and 4% iodoacetamide in equilibration buffer. The strips were then subjected to the second-dimensional electrophoresis onto 10% SDS-polyacrylamide gels. Three replicates were performed for each sample. Protein spots in the analytical gels and preparative gels were stained with coomassie brilliant blue R-250 (CBB R-250- Bio-Rad, USA) and scanned by GS-800 Calibrated Densitometer (Bio-Rad) and image analysis was accomplished using PD Quest Basic 8.0 program (Bio-Rad, USA). Each paired spot was manually verified to ensure a high level of reproducibility between normalized spot volumes of gels produced in triplicate data. The proteins of the other gels were subjected to immunoblotting assay.

### Western blot analysis

Immunoblotting of proteins separated by 2-DE was performed as demonstrated previously [24] with some modifications. Briefly, 2-DE gels were electro-blotted onto PVDF membrane (Millipore) using a trans-blot semi-dry transfer cell (Bio-Rad). Membranes were blocked with 5% dried skimmed milk and probed for 1 h at room temperature with pooled sera (1:500) from 20 infected calves in the experiment. Pooled sera collected before infection was used as a negative control. Immunoblots were established by HRP-conjugated goat anti-bovine IgG (Pierce, USA, 1:5000) for 1 h, followed by development with super signal west femto chemiluminescent substrate (Pierce, USA), and visualized on the Chemiluminescence & Fluorescence DNR Bio-imaging system (DNar Israel).

### Gel excision and tryptic digestion for MALDI-TOF MS

Protein extracts were separated and subjected to immunoblotting with experimental antisera on analytical gels. All the differentially expressed antigenic protein spots between virulent and attenuated strain of HB0801 were excised manually from the gels and digested with trypsin. In brief, gels were first treated with 200–400 µl destaining solution (100 mM NH_4_HCO_3_ in 30% ACN) and lyophilized. Extraction of peptides was performed three times with 60% ACN/0.1% TFA and freeze dried after digestion of gels in 5 µl (2.5–10 ng/µl) trypsin at 37°C overnight. The dry samples were reconstituted in 20% ACN (2 µl) and loaded on a 384-well Opti-TOF (123 mm × 81 mm) stainless steel plate. The samples in plate were then overlaid with 0.5 µl CHCA in 50% ACN and 0.1% TFA.

### MALDI-TOF MS

Mass spectrometry and MS/MS data for the recognition of proteins were generated with a MALDI-TOF appliance (4800 plus proteomics analyzer, Applied Biosystems) as described previously [24]. Collective peptide mass fingerprinting (PMF) and MS/MS inquiries were accomplished by the MASCOT search engine 2.2 (Matrix Science, Ltd., U.S.) fixed into GPS-Explorer Software 3.6 (Applied Biosystems) on NCBI database (Taxonomy: NCBI_Bacteria, number of sequences 32052081, 30/8/2013, NCBI_Mycoplasma, number of sequences 198866, 22/5/2014) and Uniprot database (Number of sequences 540732, 3/9/2013). The identified proteins sequences were obtained from NCBI. Upon comparison to cluster of orthologues groups (COGs) database using RPS-BLAST, functional classification of proteins was determined, and subcellular localization of identified proteins was anticipated with PSORTb database.

### Gel excision and tryptic digestion for LC-MS/MS

Gel pieces were cut from SDS PAGE, destained with 30% ACN/100 mM NH_4_HCO_3_ until the gels were destained. The gels were dried in a vacuum centrifuge. The in-gel proteins were reduced with dithiothreitol (10 mM DTT/100 mM NH_4_HCO_3_) for 30 min at 56°C, then alkylated with iodoacetamide (200 mM IAA/100 mM NH_4_HCO_3_) in the dark at room temperature for 30 min. Gel pieces were briefly rinsed with 100 mM NH_4_HCO_3_ and ACN, respectively. Gel pieces were digested overnight in 12.5 ng/μl trypsin in 25 mM NH_4_HCO_3_. The peptides were extracted three times with 60% ACN/0.1% TFA. The extracts were pooled and dried completely by a vacuum centrifuge.

### Liquid chromatography-tandem mass spectrometry (LC-MS/MS)

EttanTM MDLC system (GE Healthcare, Piscataway, NJ) was applied for desalting and separation of tryptic peptides mixtures. In this system, samples were desalted on RP trap columns (Zorbax 300 SB C18 peptide traps, Agilent Technologies, Wilmington, DE), and then separated on a C18 reverse-phase column (150 μm i.d., 100 mm length, Column technology Inc., Fremont, CA). Mobile phase A (0.1% formic acid in HPLC-grade water) and the mobile phase B (0.1% formic acid in acetonitrile) were selected. 20 μg of tryptic peptide mixtures were loaded onto the columns, and separation was done at a flow rate of 2 μL/min by using a linear gradient of 4–50% buffer B for 50 min, 50–100% buffer B for 4 min and 100% buffer B for 6 min. LTQ Velos (Thermo Scientific) equipped with a micro-spray interface was connected to the LC setup for eluted peptides detection. Data-dependent MS/MS spectra were obtained simultaneously. Each scan cycle consisted of one full scan mass spectrum (*m/z* 300–1800) followed by 20 MS/MS events of the most intense ions with the following dynamic exclusion settings: repeat count 2, repeat duration 30 seconds, exclusion duration 90 seconds. The LC-MS/MS analyses were repeated three times for each independent biological sample. Then the LC -MS/MS results were pooled for each biological replicate to reduce technical variation [34].

MS/MS spectra were automatically searched against the nonredundant NCBI_Mycoplasma database, number of sequences 198866, 22/5/2014 using the Bioworks Browser rev. 3.1 (Thermo Electron, San Jose, CA.). Peptide identification results were extracted from SEQUEST out files with Build Summary. The search parameters were set as: (a) trypsin digestion; (b) up to two missed cleavages allowed; (c) cysteine carbamidomethylation as a fixed modification and methionine oxidation as a variable modification; and (d) mass tolerances set at 2.0 Da for the precursor ions and 0.8 Da for fragment ones. The protein identification criteria were based on Delta CN (≥ 0.1) and cross-correlation scores (Xcorr, one charge ≥ 1.9, two charges ≥ 2.2, three charges ≥ 3.75). Only proteins identified by at least two unique peptides with good-quality tandem MS/MS data were reported.

### Quantitative real-time PCR (qRT-PCR)

Seven antigenic proteins were selected for subsequent qRT-PCR analysis based on their large numbers of predicted T- and B- cells epitopes as described previously [24] (Table 2). Total RNA was extracted using TRIzol reagent (Invitrogen, Carlbad, CA) according to the manufacturer’s instructions. Subsequently, 500 ng of the RNA was reverse transcribed into cDNA using PrimeScript RT reagent Kit (Takara, Otsu, Japan) at 37°C for 45 min, 85°C for 5 sec, 5 min at 4°C and then quantitative real-time PCR (qRT-PCR) was carried out in an ABI PRISM 7900HT Real-Time PCR system using the SYBR Premix Ex Taq II Kit (Takara) according to the manufacturer’s instructions. The thermal cycling conditions were: 2 min at 50°C, and 3 min at 95°C for initial denaturation, followed by 40 cycles of 15 sec at 95°C, 30 sec at 55°C, 30 sec at 72°C for amplification, and 15 sec at 95°C, 1 min at 55°C and 15 sec at 95°C for melting curve analysis. Target gene primers were presented in Table 3. Target gene Ct values were normalized to 16S RNA gene, and the results were analyzed by means of the 2^-∆∆Ct^ method [35].

### Site-directed mutagenesis, cloning, expression, and purification of MbovP730

The complete *M. bovis* Mbov_0730 gene was cloned and expressed in *E. coli* as demonstrated elsewhere [24] with some minor modifications. Briefly, Mbov_0730 gene was subjected to site-directed mutagenesis by overlap extension PCR primers presented in Table 4. The complete gene with the change in single nucleotide in UGA codon (UGA→UGG) was cloned into the vector pET-30a (+), confirmed by nucleotide sequencing (Sangon Company, China), expressed in *E. coli* BL21 (DE3) (Novagen, USA), and purified by nickel affinity chromatography (GE Healthcare, Sweden) as described previously (24). Protein concentration was measured by BCA method (Thermo, USA) and checked by 12% SDS-PAGE.

### Production of mouse polyclonal antibodies

BALB/c female mice (4 weeks old) were purchased from the China Hubei Provincial Center for Disease Control and Prevention, Wuhan, China and raised in the animal facility of Huazhong Agricultural University to produce polyclonal antibodies against rMbovP730 as described previously [24].

### The specificity of rMbovP730

In order to verify an absence of resultant protein (MbovP730) of gene Mbov_0730 gene in *M. bovis*-150 strain, the whole cell proteins of vaccine and virulent strains were prepared and analyzed by Western Blotting as described elsewhere [24, 36]. Briefly, 10 µg purified rMbovP730 was resolved on 12% SDS-PAGE gel and blotted on the PVDF membrane. After blocking with 5% skimmed milk overnight at 4°C, the membrane was probed for 1 h at 37°C with mouse antiserum against rMbovP730 and recombinant phosphoglycerate kinase (rPGK) as described previously [37]. After washing with PBST, plates were incubated for 1 h at 37°C with goat anti-mouse IgG-HRP (1:5000) (Southern Biotech), followed by development with super signal west femto chemiluminescent substrate (Pierce, USA), and visualized on the Chemiluminescence & Fluorescence DNr Bio-imaging system (DNr, Israel).

Additionally, antisera against IBRV (#AV20) and BVDV (#AV69) purchased from China Institute of Veterinary Drug Control were also evaluated with an iELISA to further confirm the specificity of MbovP730.

### Establishment of rMbovP730-based iELISA.

An iELISA based on rMbovP730 was established as demonstrated previously [9, 24]. Briefly, 96-well ELISA plates were coated with rMbovP730 (50 ng/well) allowed to incubate at 4°C overnight. Sera were added 1:200 (v/v). Secondary antibody, goat anti-bovine IgG-HRP, was diluted to 1:20000 (v/v). Ultimately TMB was added as substrate to the wells and incubated for 10 min, and the OD_630_ values were recorded with microplate reader. Antisera from 117 naturally infected animals and 44 immunized animals collected at various feedlots were analyzed by this iELISA for the sensitivity and specificity evaluation. The immunized calve sera were separately evaluated with assays based on the total proteins of *M. bovis*-150 and *M. bovis* HB0801. The cut-off value was determined by receiver operating characteristic (ROC) to cover 95% of population. Additionally, serum samples from 117 naturally infected animals and 20 experimentally infected animals were separately subjected to rMbovP730-based iELISA and the commercial kit (BioX, Belgium) under the standardized conditions, and the degree of agreement and sensitivity between the two assays were determined.

### Statistical analysis

Each experiment was repeated at least three times independently. Data were expressed as mean ± SD and evaluated with Student’s *t*-test, and 2-way ANOVA. Determination of cut-off value, sensitivity and specificity based on ROC analysis were determined online by using EpiTools^1^. *p* < 0.05 and *p* < 0.01 were considered statistically significant (^*^) and very significant (^**^).

## References

[1] Razin S, Yogev D, Naot Y (1998). Molecular biology and pathogenicity of mycoplasmas. Microbiol Mol Biol Rev.

[2] Nicholas RA, Ayling RD (2003). Mycoplasma bovis: disease, diagnosis and control. Res Vet Sci.

[3] Hermeyer K, Buchenau I, Thomasmeyer A, Baum B, Spergser J, Rosengarten R, Hewicker-Trautwein M (2012). Chronic pneumonia in calves after experimental infection with Mycoplasma bovis strain 1067: characterization of lung pathology, persistence of variable surface protein antigens and local immune response. Acta Vet Scand.

[4] Mustafa R, Qi J, Ba X, Chen Y, Hu C, Liu X, Tu L, Peng Q, Chen H, Guo A (2013). *In vitro* Quinolones Susceptibility Analysis of Chinese Mycoplasma bovis Isolates and their Phylogenetic Scenarios based upon QRDRs of DNA Topoisomerases Revealing a Unique Transition in ParC. Pak Vet J.

[5] Zhang R, Han X, Chen Y, Mustafa R, Qi J, Chen X, Hu C, Chen H, Guo A (2014). Attenuated Mycoplasma bovis strains provide protection against virulent infection in calves. Vaccine.

[6] Lei S, Rui G, Zhengyan Y, Yong Z, Jie P, Zhibin H, Lixia W, Changmin H, Tao L, Yingyu C, Juanhong L, Junlong Z, Huanchun C, Aizhen G (2008). Diagnosis of Cattle Infectious Mycoplasma bovis pneumonia. J Huazhong Agri Uni.

[7] Caswell JL, Archambault M (2007). Mycoplasma bovis pneumonia in cattle. Anim Health Res Rev.

[8] Qi J, Guo A, Cui P, Chen Y, Mustafa R, Ba X, Hu C, Bai Z, Chen X, Shi L, Chen H (2012). Comparative geno-plasticity analysis of Mycoplasma bovis HB0801 (Chinese isolate). PLoS One.

[9] Han X, Khan FA, Zhu X, Zhang R, Mustafa R, Hu C, Chen Y, Chen H, Guo A (2015). Establishment of an antibody avidity test to differentiate vaccinated cattle from those naturally infected with Mycoplasma bovis. Vet J.

[10] Wise KS, Calcutt MJ, Foecking MF, Röske K, Madupu R, Methé BA (2011). Complete genome sequence of Mycoplasma bovis type strain PG45 (ATCC 25523). Infect Immun.

[11] Li Y, Zheng H, Liu Y, Jiang Y, Xin J, Chen W, Song Z (2011). The complete genome sequence of Mycoplasma bovis strain Hubei-1. PLoS One.

[12] Regula JT, Ueberle B, Boguth G, Görg A, Schnölzer M, Herrmann R, Frank R (2000). Towards a two-dimensional proteome map of Mycoplasma pneumoniae. Electrophoresis.

[13] Browning GF, Marenda MS, Noormohammadi AH, Markham PF (2011). The central role of lipoproteins in the pathogenesis of mycoplasmoses. Vet Microbiol.

[14] Adamu JY, Wawegama NK, Browning GF, Markham PF (2013). Membrane proteins of Mycoplasma bovis and their role in pathogenesis. Res Vet Sci.

[15] Jores J, Meens J, Buettner FF, Linz B, Naessens J, Gerlach GF (2009). Analysis of the immunoproteome of Mycoplasma mycoides subsp. mycoides small colony type reveals immunogenic homologues to other known virulence traits in related Mycoplasma species. Vet Immunol Immunopathol.

[16] Sun Z, Fu P, Wei K, Zhang H, Zhang Y, Xu J, Jiang F, Liu X, Xu W, Wu W (2014). Identification of novel immunogenic proteins from Mycoplasma bovis and establishment of an indirect ELISA based on recombinant E1 beta subunit of the pyruvate dehydrogenase complex. PLoS One.

[17] Dallo SF, Kannan TR, Blaylock MW, Baseman JB (2002). Elongation factor Tu and E1 beta subunit of pyruvate dehydrogenase complex act as fibronectin binding proteins in Mycoplasma pneumoniae. Mol Microbiol.

[18] Perez-Casal J, Prysliak T (2007). Detection of antibodies against the Mycoplasma bovis glyceraldehyde-3-phosphate dehydrogenase protein in beef cattle. Microbial Pathogenesis.

[19] Prysliak T, Van-der-Merwe J, Perez-Casal J (2013). Vaccination with recombinant Mycoplasma bovis GAPDH results in a strong humoral immune response but does not protect feedlot cattle from an experimental challenge with M. bovis. Microbial Pathogenesis.

[20] Pittau M, Fadda M, Briguglio P, Farina S, Carboni AQ, Contini A (1990). Triton X-114 phase fractionation of Mycoplasma agalactiae membrane proteins and affinity purification of specific antibodies. Atti Soc Ital Sci Vet.

[21] Bordier C (1981). Phase separation of integral membrane proteins in Triton X-114 solution. J Biol Chem.

[22] Cacciotto C, Addis MF, Pagnozzi D, Chessa B, Coradduzza E, Carcangiu L, Uzzau S, Alberti A, Pittau M (2010). The liposoluble proteome of Mycoplasma agalactiae: an insight into the minimal protein complement of a bacterial membrane. Bmc Microbiol.

[23] Parraga-Nino N, Colome-Calls N, Canals F, Querol E, Ferrer-Navarro M (2012). A Comprehensive Proteome of Mycoplasma genitalium. J. Proteome Res.

[24] Khan FA, Faisal M, Chao J, Liu K, Chen X, Zhao G, Menghwar H, Zhang H, Zhu X, Rasheed MA, He C, Hu C, Chen Y (2016). Immunoproteomic identification of MbovP579, a promising diagnostic biomarker for serological detection of Mycoplasma bovis infection. Oncotarget.

[25] Khan FA, Rasheed MA, Faisal M, Menghwar H, Zubair M, Sadique U, Chen H, Guo A (2017). Proteomics analysis and its role in elucidation of functionally significant proteins in Mycoplasma bovis. Microb Pathog.

[26] Chambaud I, Wróblewski H, Blanchard A (1999). Interactions between mycoplasma lipoproteins and the host immune system. Trends Microbiol.

[27] Wawegama NK, Browning GF, Kanci A, Marenda MS, Markham PF (2014). Development of a recombinant protein-based enzyme-linked immunosorbent assay for diagnosis of Mycoplasma bovis infection in cattle. Clin Vaccine Immunol.

[28] Behrens A, Poumarat F, Le Grand D, Heller M, Rosengarten R (1996). A newly identified immunodominant membrane protein (pMB67) involved in Mycoplasma bovis surface antigenic variation. Microbiology.

[29] Sachse K, Helbig JH, Lysnyansky I, Grajetzki C, Müller W, Jacobs E, Yogev D (2000). Epitope mapping of immunogenic and adhesive structures in repetitive domains of Mycoplasma bovis variable surface lipoproteins. Infect Immun.

[30] Citti C, Nouvel LX, Baranowski E (2010). Phase and antigenic variation in mycoplasmas. Future Microbiol.

[31] Herwig S, Kruft V, Wittmann-Liebold B (1992). Primary structures of ribosomal proteins L3 and L4 from Bacillus stearothermophilus. Eur J Biochem.

[32] Rasheed MA, Qi J, Zhu X, Chenfei H, Menghwar H, Khan FA, Zhao G, Zubair M, Hu C, Chen Y, Chen H, Guo A (2017). Comparative Genomics of Mycoplasma bovis Strains Reveals That Decreased Virulence with Increasing Passages Might Correlate with Potential Virulence-Related Factors. Front Cell Infect Microbiol.

[33] Menghwar H, Chenfei H, Hui Z, Gang Z, Xifang Z, Khan FA, Faisal M, Rasheed MA, Zubair M, Memon AM, Ridley A, Robertson ID, Yingyu C, Aizhen G (2017). Genotype distribution of Chinese Mycoplasma bovis isolates and their evolutionary relationship to strains from other countries. Microbial Pathogenesis.

[34] Donoghue PM, Hughes C, Vissers JP, Langridge JI, Dunn MJ (2008). Nonionic detergent phase extraction for the proteomic analysis of heart membrane proteins using label-free LC-MS. Proteomics.

[35] Livak KJ, Schmittgen TD (2001). Analysis of relative gene expression data using real-time quantitative PCR and the 2(−Delta Delta C (T)) Method. Methods.

[36] Guo Y, Zhu H, Wang J, Huang J, Khan FA, Zhang J, Guo A, Chen X (2017). TrmFO, a Fibronectin-Binding Adhesin of Mycoplasma bovis. Int J Mol Sci.

[37] Zhao G, Zhang H, Chen X, Zhu X, Guo Y, He C, Anwar Khan F, Chen Y, Hu C, Chen H, Guo A (2017). Mycoplasma bovis NADH oxidase functions as both a NADH oxidizing and O(2) reducing enzyme and an adhesin. Sci Rep.

